# Effects of Ranolazine on Astrocytes and Neurons in Primary Culture

**DOI:** 10.1371/journal.pone.0150619

**Published:** 2016-03-07

**Authors:** Martin Aldasoro, Sol Guerra-Ojeda, Diana Aguirre-Rueda, Mª Dolores Mauricio, Jose Mª Vila, Patricia Marchio, Antonio Iradi, Constanza Aldasoro, Adrian Jorda, Elena Obrador, Soraya L. Valles

**Affiliations:** Department of Physiology, School of Medicine, University of Valencia, Spain; University of Louisville, UNITED STATES

## Abstract

Ranolazine (Rn) is an antianginal agent used for the treatment of chronic angina pectoris when angina is not adequately controlled by other drugs. Rn also acts in the central nervous system and it has been proposed for the treatment of pain and epileptic disorders. Under the hypothesis that ranolazine could act as a neuroprotective drug, we studied its effects on astrocytes and neurons in primary culture. We incubated rat astrocytes and neurons in primary cultures for 24 hours with Rn (10^−7^, 10^−6^ and 10^−5^ M). Cell viability and proliferation were measured using trypan blue exclusion assay, MTT conversion assay and LDH release assay. Apoptosis was determined by Caspase 3 activity assay. The effects of Rn on pro-inflammatory mediators IL-β and TNF-α was determined by ELISA technique, and protein expression levels of Smac/Diablo, PPAR-γ, Mn-SOD and Cu/Zn-SOD by western blot technique. In cultured astrocytes, Rn significantly increased cell viability and proliferation at any concentration tested, and decreased LDH leakage, Smac/Diablo expression and Caspase 3 activity indicating less cell death. Rn also increased anti-inflammatory PPAR-γ protein expression and reduced pro-inflammatory proteins IL-1 β and TNFα levels. Furthermore, antioxidant proteins Cu/Zn-SOD and Mn-SOD significantly increased after Rn addition in cultured astrocytes. Conversely, Rn did not exert any effect on cultured neurons. In conclusion, Rn could act as a neuroprotective drug in the central nervous system by promoting astrocyte viability, preventing necrosis and apoptosis, inhibiting inflammatory phenomena and inducing anti-inflammatory and antioxidant agents.

## Introduction

Ranolazine (Rn), a piperazine derivative, is indicated for the treatment of refractory chronic stable angina, in combination with other anti-ischemic drugs [1,2]. In contrast to other agents, Rn does not significantly modify systemic blood pressure or heart rate [3,4]. Rn produces cardiovascular benefits by inhibiting the late inward sodium current (late *I*_Na_) [5]. Late *I*_Na_ amplitude is increased in many pathological situations, such as myocardial ischemia and oxidative stress [6,7]. At therapeutic concentrations, Rn inhibits *I*_Ca_ channels [8] and the slowly inactivating components of the sodium current (late *I*_Na_), reducing tissue damage caused by intracellular sodium and calcium overload associated with myocardial ischemia [9,10].

In addition to its antianginal effects, Rn acts as an anti-inflammatory agent by reducing asymmetric dimethylarginine and C-reactive protein plasma levels, and by promoting endothelial release of vasodilator mediators in patients with ischemic coronary disease [11]. Furthermore, it has been described metabolic effects, such as the lowering of hemoglobin A1C (HbA1c) in patients with ischemic heart disease and diabetes [12], or the improvement of insulin secretion and β-cell survival in diabetic mice [13].

Moreover, several studies have evaluated the effects of Rn on central nervous system (CNS). By decreasing neuronal excitability, Rn acts as an anticonvulsant agent [14,15], and has been proposed as a possible treatment for neuropathic pain [16]. It has been suggested that these effects would be mediated by late *I*_Na_ or inwardly rectifying K^+^ current [17], enabling the development of new treatment strategies for chronic pain [18], or epileptic disorders [19].

Among neural cells, astrocytes play different roles, such as structural support and maintenance of the blood brain integrity [20], and are involved in immunological responses and reparative processes that occur at different stages of neuroinflammation [21]. Astrocytes produce both neurotropic and inflammatory cytokines, and express receptors for mediators such as IL-1β, TNF-α, among others [22,23]. We have recently determined, using mixed glial-neuronal cultures, that astrocytes protect neurons from toxic agents, such as Aβ_1–42_, through mitochondrial biogenesis increase [24], and that some drugs can increase cell viability and anti-inflammatory response in astrocytes, preventing cell death and inflammation induced by Aβ_1–42_ [25].

Therefore, the purpose of this study is to investigate the effects of Rn on cell viability, apoptosis, inflammation and oxidative stress in astrocytes and neurons in primary culture.

## Material and Methods

### Materials

The study was approved by the Bioethics Committee of the School of Medicine of the University of Valencia, Spain (number A1265026030697). All animals were handled according to the recommendations of the Committee. Dulbecco’s modified Eagle’s medium (DMEM) and foetal bovine serum (FBS) were obtained from Gibco (Gibco Invitrogen Corporation, Barcelona, Spain). Ranolazine (Rn) was obtained from Sigma-Aldrich biotechnology and dissolved in Krebs solution to the proper final concentration (10^−7^, 10^−6^, 10^−5^ M). 3-(4,5-dimethyl-2-thiazolyl)-2,5-dipheniyl-2H-tetrazolium bromide (MTT) was purchased from Sigma Chemical Co. (St Louis, MO). Enzyme-linked immunosorbent assay (ELISA) kits for IL-1β and TNF-α from Pierce Biotechnology, Inc. (Rockford, USA). Western Blot Chemiluminescent Detection System (ECL) was from Amersham (Amersham Biosciences, Barcelona, Spain). Monoclonal anti-peroxisome proliferator-activated receptor antibody (PPAR-γ) (1:250) from Sigma Aldrich (Madrid, Spain). Monoclonal anti-Smac/Diablo antibody (1:250) from Santa Cruz Biotechnology (Madrid, Spain). Polyclonal anti-Cu/Zn superoxide dismutase antibody (Cu/Zn-SOD) (1:250) from Assay Designs (Madrid, Spain). Monoclonal anti-Mn superoxide dismutase (1:500) and anti-tubuline (1:1000) antibodies from Cell Signaling (Beverly, MA, USA). Anti-GFAP (1:1000) and anti-MAP-2 (1:1000) antibodies from Sigma Aldrich (Barcelona, Spain). Amyloid β_1–42_ was diluted to 100 μM in phosphate buffered saline (PBS), and the oligomer preparations were aged for 24 h at 37°C, following manufacturer´s instruction (Sigma-Aldrich, Barcelona, Spain). All other reagents are analytical or culture grade purity.

### Primary Culture of Cortical Astrocytes

Cerebral cortical astrocytes were isolated from rat foetuses of 21 days gestation. Foetuses were obtained by cesarean section and decapitated. Cerebral cortices were removed and triturated 10–15 times through a Pasteur pipette. The cell suspension was filtered through nylon mesh with a pore size of 90 μm and was diluted in DMEM containing 20% foetal bovine serum (FBS) supplemented with L-glutamine (1%), HEPES (10 mM), fungizone (1%), and antibiotics (1%). Cells were plated on T75 culture flask pretreated with poli-L-lysine. Cultures were maintained in a humidified atmosphere of 5% CO2/95% air at 37°C during 20 days. After 1 week of culture, the FBS content was reduced to 10%, and the medium was changed twice a week. By immunocytochemistry, 97% of cells are GFAP positive (data not shown).

### Primary Culture of Cortical Neurons

Primary cultures of rat cortical neurons were prepared from the cerebral cortex of 14-day old rat foetuses. Briefly, the cerebral cortex of foetuses obtained under sterile conditions were dissected and dissociated mechanically, by pipetting 10 times with 10 ml of DMEM (Gibco Invitrogen Corporation, Barcelona, Spain). The cell suspension was filtered through nylon mesh with a pore size of 90 μm. Cell suspension was plated (5 x 104 cells/cm2) on poly- Lysine-coated dishes. After attachment of the cells, the plating medium was changed to DMEM containing 10% FBS supplemented with antibiotics (1%) and fungizone (1%). Cultures were grown in a humidified atmosphere of 5% CO2/95% air at 37°C for 3 days. Cells were then exposed to 10 μM cytosine β-D arabino-furanoside on the third day of culture for 24 h to inhibit proliferation of non-neuronal cells. The medium was changed twice a week. By immunocytochemistry, 98% of cells are neurons (MAP-2 positive) (data not shown).

### Trypan Blue Assay

Trypan blue exclusion assay was used to count the living cells and monitor cell proliferation. Astrocytes were isolated and seeded at 7x10^4^ cells/35 mm dish. After 5 days of culture, cells were incubated without (control, C) or with Rn (10^−7^, 10^−6^, 10^−5^ M) for 24 h. 1.5% trypan blue solution was applied to astrocytes cultures at room temperature for 3 min.

### MTT Assay

Cell viability of the cultures was determined by the MTT assay. Cells were plated in 96 well culture plate and incubated with Rn during 24 h at different concentrations (10^−7^, 10^−6^ and 10^−5^ M). After cell treatments, the medium was removed and the cortical cells were incubated with red free medium and MTT solution [0.5 mg/ml, prepared in phosphate buffer saline (PBS) solution] for 4 h at 37°C. Finally the medium was removed and formazan particles were dissolved in dimethyl sulfoxide (DMSO). Cell viability, defined as the relative amount of MTT reduction, was determined by spectrophotometry at 570 nm.

### Lactate Dehydrogenase (LDH) Assay

To evaluate plasma membrane integrity, LDH release was determined by monitoring the leakage of the cytosolic LDH to the extracellular medium). LDH was measured spectrophotometrically at 340 nm, following the rate of conversion of reduced nicotinamide adenine dinucleotide to oxidized nicotinamide adenine dinucleotide.

### Caspase 3 Activity Assay

Caspase 3 activity was measured in cytosolic fractions by using a highly sensitive colorimetric substrate, N-acetyl-Asp-Glu-Val-Asp p-nitroanilide (Ac-DEVD-pNA) following manufacturer´s instructions (CalBiochem, La Jolla, CA). Enzyme activity was calculated using manufacturer´s formulae, as pmol/min.

### Cytokine Determination, IL-1 and TNFα

Cells were seeded, and at time of assay, the red phenol medium was removed and replaced by PBS containing 1 mg/ml bovine serum albumin (BSA), either in the presence or absence of Rn at different concentrations (10^−7^, 10^−6^ and 10^−5^ M). IL-1β and TNF-α concentration (pg/ml) were ascertained using ELISA kits (Pierce Biotechnology, Inc.).

### Western Blot Analysis

Cultured cells were treated with lysis buffer and then mechanically degraded to release the proteins. Protein concentration was determined using modified Lowry method [26]. Loading buffer (0.125 M Tris-HCl, pH 6.8, 2% SDS, 0.5% (v/v) 2-mercaptoethanol, 1% bromophenolblue and 19% glycerol) was added to protein sample and heated for 5 min at 95°C. Proteins (20 μg) were separated on SDS-PAGE gels and transferred to nitrocellulose membranes in a humid environment using a transfer buffer (25 mM Tris, 190 mM glycine, 20% methanol). Membranes were blocked with 5% milk in TBS-T (0.05% Tween-20) and incubated with primary antibodies overnight at 4°C. Membranes were washed 3 times with wash buffer TBS-T (TBS, 0.2% Tween-20) and incubated with a secondary anti-rabbit IgG or anti-mouse IgG (Cell Signalling Technologies Danvers, MA) antibody conjugated to the enzyme horseradish peroxidase (HRP) for 1 h. Membranes were washed three times and proteins were detected using the ECL method as specified by the manufacturer. Autoradiography signals were assessed using digital image system ImageQuant LAS 4000 (GE Healthcare). Densitometry is the quantitative measurement of optical density in a photographic paper or photographic film, due to exposure to light. Concentration of protein was determined by densitometric analysis, expressed as arbitrary units or relative densitometric units, relative to tubulin.

### Statistical Analyses

Values are expressed as mean ± S.D. Statistical analysis were performed in two steps. First, an analysis of variance was performed. Second, the sets of data in which *F* was significant were performed using *t*-test (Student´s t test). Differences between groups were assessed by one-way analysis of variance (ANOVA) performed with the program GraphPad Prism. Statistical significance was accepted at *p* ≤ 0.05.

## Results

### Rn and Cell Viability

Trypan blue exclusion assay was used to count the living cells and monitor cell proliferation. Astrocytes were isolated and seeded at 7x10^4^ cells/35 mm dish. After 5 days of culture, cells were incubated without (control, C) or with Rn (10^−7^, 10^−6^, 10^−5^ M) for 24 h. In control conditions proliferation was 0.9%, and previous incubation with Rn increased proliferation by 15% (10^-7^M), 37% (10^-6^M) and 39% (10^-5^M) respectively (Table 1).

**Table 1:** Astrocytes proliferation and counting living cells. Astrocytes were isolated and seeded at 7x10^4^ cells/35 mm dish during 5 days. At this time, cells were incubated without Rn (control, C) or with Rn (10–7, 10–6, 10–5 M) for 24 h. Trypan blue exclusion was used to count the living cells and monitor cell proliferation. Data are mean ± SD of four independent experiments (four different rats).

	Seeding cells (x10^4^/35 mm dish)	5 days of culture	Rn 24h (x10^4^/35 mm dish)	% Proliferation
**Control**	7	12.47 ± 0.21	12.58 ± 0.18	0.9
**Rn 10**^**-7**^**M**	7	12.25 ± 0.32	14.09 ± 0.26 *	15
**Rn 10**^**-6**^**M**	7	12.18 ± 0.45	16.69 ± 0.25 *	37
**Rn 10**^**-5**^**M**	7	12.31 ± 0.51	17.11 ± 0.31 *	39

*p < 0.05 vs. control.

**Fig 1:**
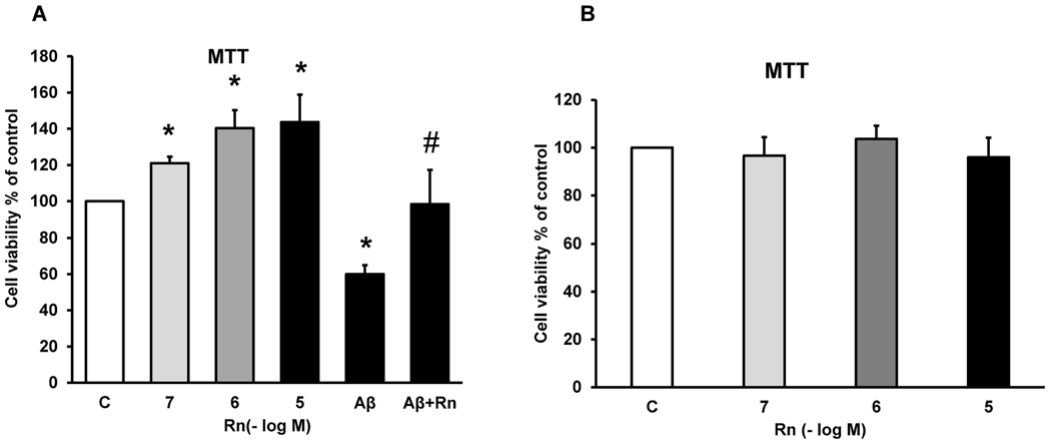
Cell viability was determined by MTT assay in cells treated for 24 h. Astrocytes were incubated without Rn (control, C), with Rn (10^−7^, 10^−6^, 10^−5^ M), with Amyloid β_1–42_ (15 μM) or with Amyloid β_1–42_ (15 μM) + Rn (10^−6^ M) (panel A). Neurons were incubated without (control, C) or with Rn (10^−7^, 10^−6^, 10^−5^ M) (panel B). Data are mean ± SD of four independent experiments (four different rats). *p < 0.05 *vs*. control.

The role of Rn on cell viability was also studied using MTT conversion assay. Fig 1A shows that incubation with Rn at different concentrations (10^−7^, 10^−6^ and 10^−5^ M), induced a significant increase in astrocytes viability at any concentration tested (21% (10^-7^M), 40% (10^-6^M) and 43% (10^-5^M)) compared with control cells.

Aβ_1–42_ (15μM) significantly decreased cell viability compared to control astrocytes (38%). Incubation with Rn (10^-6^M) and the toxic peptide prevented the decrease in cell viability induced by Aβ_1–42_.

**Fig 2:**
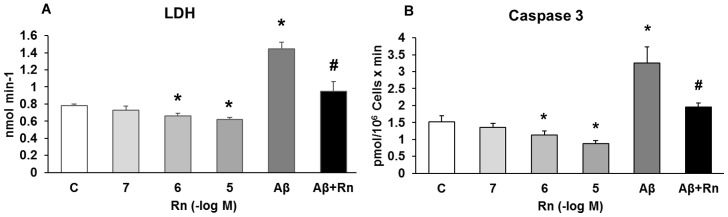
Lactate dehydrogenase and caspase 3 activity. Astrocytes were incubated without Rn (control, C), with Rn (10^−7^, 10^−6^, 10^−5^ M), with Amyloid β_1–42_ (15 μM) or with Amyloid β_1–42_ (15 μM) + Rn (10^−6^ M) for 24 h. Panel A: Lactate dehydrogenase activity was measured in supernatants of astrocytes. Panel B: Caspase 3 activity were determined as indicated under Materials and Methods. Data are means ± standard deviation of three independent experiments (three different rats). *p < 0.05 versus control cells. #*p* < 0.05 versus Aβ-treated cells.

Neurons previously incubated with Rn (10^−7^, 10^−6^ and 10^−5^ M) for 24 h showed no differences in cell viability compared to control cells (Fig 1B). Incubation of astrocytes with Rn (10^−6^ and 10^−5^ M) for 24 h decreased LDH (15% with Rn 10^-6^M and 20% with Rn 10^-5^M) release to the medium and prevented LDH leakage induced by Aβ_1–42_ (15μM)_,_ indicating that astrocytes are protected in some way by Rn (Fig 2A). Toxic peptide increased LDH release in about 75% and incubation with Rn (10^-6^M) lowered by 60% LDH levels, indicating a protective effect against Aβ_1–42_.

### Caspase 3 and Smac/Diablo Expression

Incubation with Rn (10^−6^ and 10^−5^ M) for 24 h decreased Caspase 3 activity to 25% (Rn 10^-6^M) and to 40% (Rn 10^-5^M), compared to control cells whereas activity was increased by treatment with Aβ_1–42_ (15 μM) (Fig 2B). In the culture medium Aβ_1–42_ increased caspase-3 activity (105%), that was reversed by Rn in 85%, (Fig 2B), indicating reduction of apoptosis after Rn addition to the culture.

**Fig 3:**
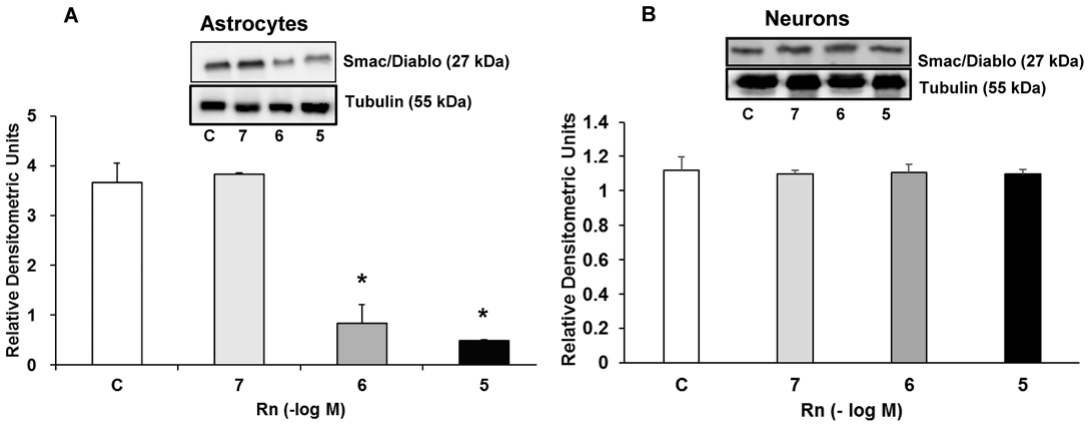
Smac/Diablo protein expression. Astrocytes (panel A) or neurons (panel B) were incubated without Rn (control, C) or with Rn (10^−7^, 10^−6^, 10^−5^ M) for 24 h and collected to determine Smac/Diablo protein expression by Western blot. A representative immunoblot is shown in the top panel. Data are mean ± SD of four independent experiments (four different rats). *p < 0.05 vs. control.

Fig 3 shows Smac/Diablo expression in astrocytes and neurons in culture. In astrocytes, Rn decreased Smac/Diablo expression (4.4-fold at 10^−6^ M and 7.6-fold at 10^−5^ M) (Fig 3A). On the other hand, we did not find any difference in cultured neurons compared to control cells at all concentrations used (10^−7^, 10^−6^ and 10^−5^ M) (Fig 3B).

### Rn and IL-1β Pro-Inflammatory Cytokine

**Fig 4:**
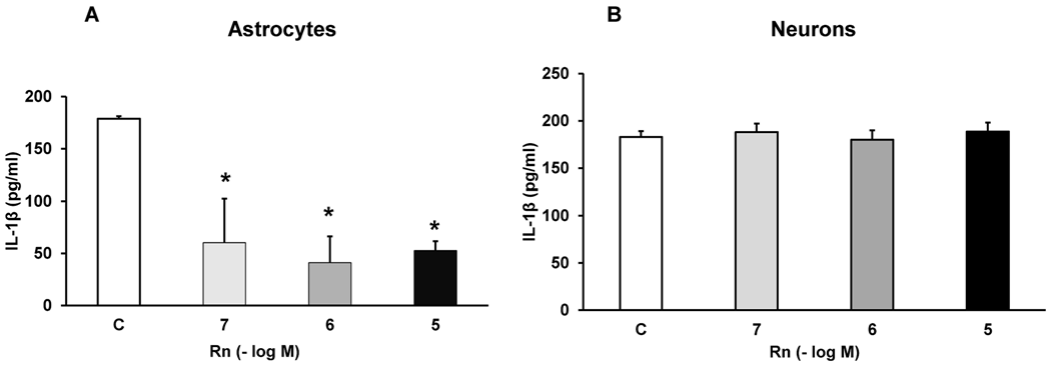
Cytokine IL-1β determination. Astrocytes (panel A) or neurons (panel B) were incubated without Rn (control, C) or with Rn (10^−7^, 10^−6^, 10^−5^ M) and cell culture supernatants were harvested. IL-1β secretion were determined by ELISA. Values are means ± SD of replicate experiments from four independent experiments (four different rats). *p < 0.05 *vs* control.

Cultured astrocytes and neurons were incubated with Rn (10^−7^, 10^−6^ and 10^−5^ M) and secretion of IL-1β was detected by ELISA. Fig 4 shows that, in astrocytes, Rn decreased 3.65, 4.14 and 4.53-fold IL-1β release at 10^−7^, 10^−6^ and 10^−5^ M respectively, compared with control values (Fig 4A). However, Rn did not change IL-1β release in neurons at any concentration tested (Fig 4B).

### Rn and TNF-α Pro-Inflammatory Mediator

**Fig 5:**
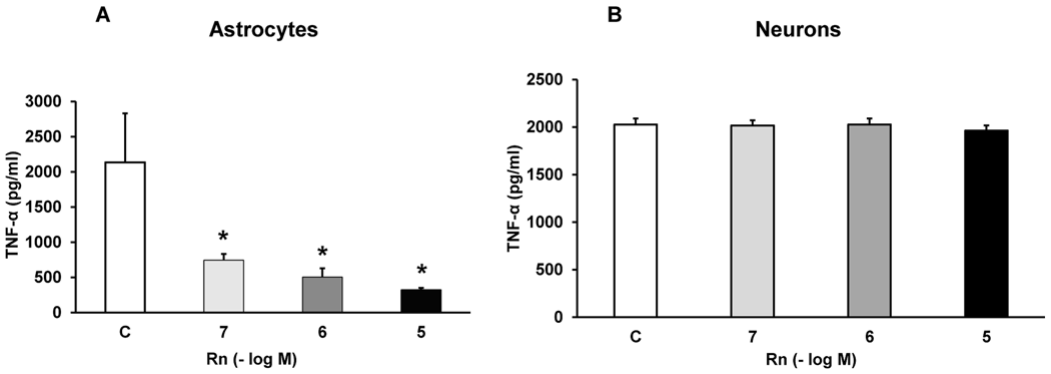
Cytokine TNF-α determination. Astrocytes (panel A) or neurons (panel B) were incubated without Rn (control, C) or with Rn (10^−7^, 10^−6^, 10^−5^ M) and cell culture supernatants were harvested. TNF-α secretion were determined by ELISA. Values are means ± SD of replicate experiments from four independent cell experiments (four different rats). *p < 0.05 *vs* control.

TNF-α levels were detected by ELISA. Fig 5 shows the effects elicited by Rn (10^−7^, 10^−6^ and 10^−5^ M) on astrocytes and neurons in primary culture. In astrocytes, Rn decreased 2.87, 4.21 and 6.63-fold TNF-α release at 10^−7^, 10^−6^ and 10^−5^ M respectively, compared to control cells (Fig 5A). On the contrary, Rn did not induce any change in cultured neurons (Fig 5B).

### Rn and PPAR-γ Expression

**Fig 6:**
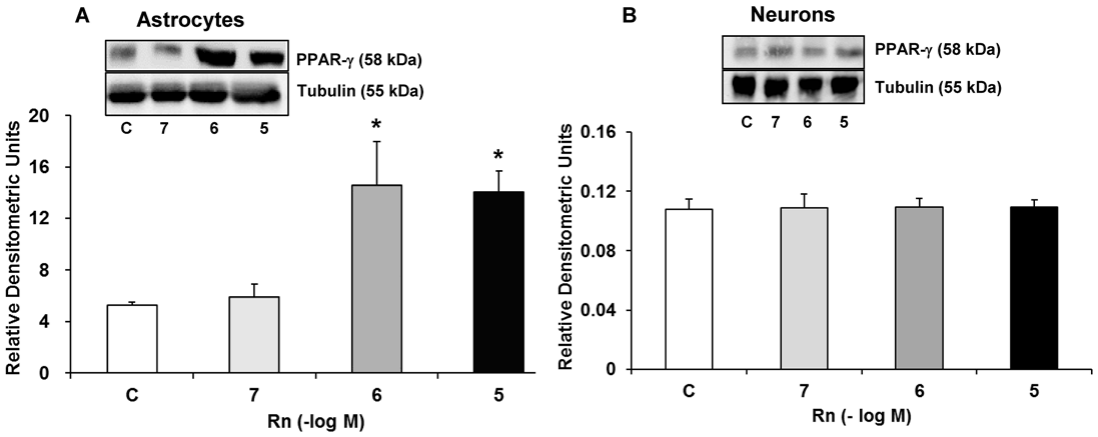
PPAR-γ protein expression. Astrocytes (panel A) or neurons (panel B) were incubated without Rn (control, C) or with Rn (10^−7^, 10^−6^, 10^−5^ M) for 24 h and collected to determine PPAR-γ protein expression by Western blot. A representative immunoblot is shown in the top panel. Data are mean ± SD of four independent experiments (four different rats). *p < 0.05 vs. control.

PPARs family regulates negatively gene expression of pro-inflammatory proteins. (Fig 6A and 6B) shows PPAR-γ expression in astrocytes and neurons in culture. Rn increased astrocytes PPAR-γ expression 2.72-fold at 10^−6^ M and 2.84-fold at 10^−5^ M compared to control cells (Fig 6A). When Rn was added to cultured neurons, no changes in PPAR-γ expression were detected at any concentration analyzed compared to controls (Fig 6B).

### Rn and Cu/Zn-SOD Expression

**Fig 7:**
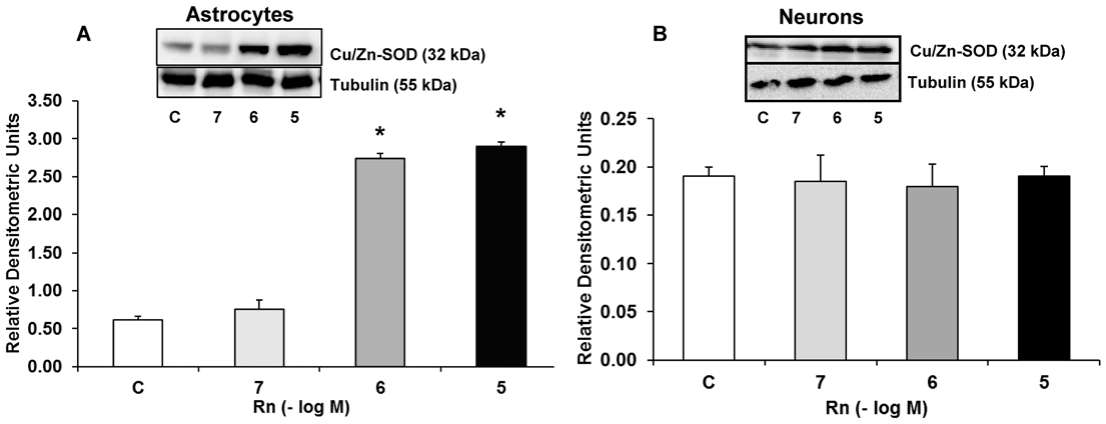
Cu/Zn-SOD protein expression. Astrocytes (panel A) or neurons (panel B) were incubated without Rn (control, C) or with Rn (10^−7^, 10^−6^, 10^−5^ M) for 24 h and collected to determine Cu/Zn-SOD protein expression by Western blot. A representative immunoblot is shown in the top panel. Data are mean ± SD of four independent experiments (four different rats). *p < 0.05 vs. control.

Fig 7 shows Cu/Zn-SOD expression in astrocytes and neurons in primary culture. In astrocytes, Rn increased Cu/Zn-SOD expression 4.49-fold at 10^−6^ M and 4.74-fold at 10^−5^ M compared to control cells (Fig 7A). We also analyzed Rn effects in neurons, and we did not detect differences in Cu/Zn-SOD expression compared to control cells at any concentration tested (Fig 7B).

### Rn and Mn-SOD Expression

**Fig 8:**
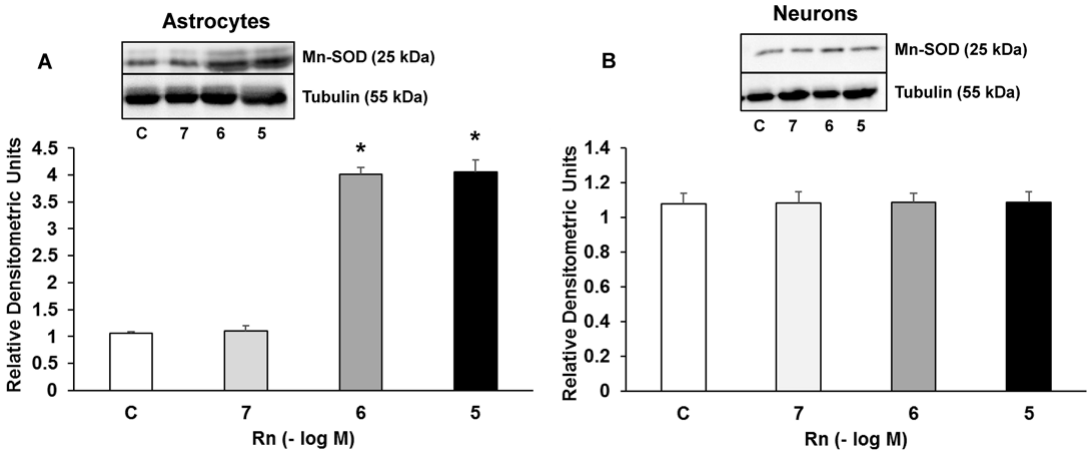
Mn-SOD protein expression. Astrocytes (panel A) or neurons (panel B) were incubated without Rn (control, C) or with Rn (10^−7^, 10^−6^, 10^−5^ M) for 24 h and collected to determine Mn-SOD protein expression by Western blot. A representative immunoblot is shown in the top panel. Data are mean ± SD of four independent experiments (four different rats). *p < 0.05 vs. control.

Fig 8 shows Mn-SOD expression in astrocytes and neurons in primary culture. In astrocytes, Rn (10^−6^, 10^−5^ M) increased Mn-SOD expression 4.12-fold and 4.20-fold respectively compared to control cells (Fig 8A). We also analyzed Rn effects in neurons and we did not detect differences in Mn-SOD compared to control cells at any concentration tested (Fig 8B).

## Discussion

The main findings of this study are that Rn decreased pro-inflammatory mediators IL-1β and TNF-α release, and increased anti-inflammatory PPAR-γ as well as the antioxidant enzymes Cu/Zn-SOD and Mn-SOD expressions in primary culture of astrocytes. Furthermore, Rn increased astrocytes viability and proliferation and reduced LDH release, caspase 3 activity and apoptosis activity via Smac/Diablo protein. On the other hand, we did not observe any effect of Rn on neurons in primary culture.

Astrocytes are specialized neural cells playing different roles in the CNS, such as structural and metabolic support to the brain, synthesis of glutathione and its precursors, brain trophic role [21,27], and neuron protection against oxidative stress and inflammation [24,28,29]. The exact mechanisms by which astrocytes protect neurons remain to be determined.

Anti-inflammatory substances can act in some glial cells [30]. Rn can cross blood brain barrier, reaching in brain about one-third of plasma levels [19]. Rn acts as an anti-inflammatory agent by reducing asymmetric dimethylarginine and C-reactive protein plasma levels [11], significantly reducing infarct size [31]. At therapeutic concentrations, Rn blocks voltage-gated sodium channels (VGSCs), preferentially the late sodium current (*I*_NaL_) [32]. Sodium channel inhibitors exert neuro-protective effects in experimental models of brain ischemia [33] and traumatic brain injury [34]. Moreover, phenytoin and carbamazepine were effective in animal models of autoimmune encephalomyelitis (EAE) [35]. Additionally, tetrodotoxin attenuated astrogliosis induced by an increase in voltage-gated sodium channels in an *in vitro* rat model of mechanical injury [36].

Reactive astrogliosis occurs in response to CNS insults inducing changes in many astrocyte functions, including oxidative stress and inflammation, contributing to CNS lesions [23]. Our study demonstrates that Rn decreased pro-inflammatory cytokines TNF-α and IL-1β release in cultured astrocytes. In normal conditions, there is a balance between pro- and anti-inflammatory cytokines in order to maintain cell equilibrium. Most cytokines at very low concentrations regulate cellular activities, including cell survival, growth, and differentiation [37]. Astrocytes play a major role in neuro-inflammation and, depending on the stimulus, location and time course of the insult, reactive astrocytes can exert both pro- and anti-inflammatory effects [23,37]. Reactive astrogliosis [38] can be triggered by various inflammatory mediators such as TNF-α and IL-1β [23,39]. Prolonged or uncontrolled inflammation results detrimental, exacerbating neural damage by overexpression of pro-inflammatory factors that enhance inflammation through a positive feedback loop, inducing the production of more cytokines or reactive oxygen species (ROS), among other deleterious effects [23,37,39,40,41]. Pro-inflammatory cytokines may increase neuronal excitability by releasing glutamate from astrocytes by a Ca^2+^-dependent mechanism, resulting in excitotoxic injury to neurons, and also induce apoptosis in neurons and glial cells [42]. In this sense, TNF-α may directly elicit apoptosis through receptor TNF-R1 whereas apoptosis induced by IL-1 depends on other mediators such as IFN-γ or TNF-α. Pro-inflammatory cytokines can also provoke an increased production of neurotoxic factors such as ROS and nitric oxide (NO) [37]. Elevated concentrations of pro-inflammatory cytokines, including IL-1β and TNF-α, were found in blood, CSF and other tissues in Alzheimer and Parkinson diseases, amyotrophic lateral sclerosis and severe subarachnoid hemorrhage, evidencing the participation of inflammation in the pathogenesis and outcome of these diseases [37,40,43]. TNF-α can also induce the expression of other cytokines, such as IL-1, and both have the capacity to induce IL-6, a marker for systemic inflammatory responses [44]. Sodium channel blockade attenuates the release of pro-inflammatory cytokines IL-1β and TNF-α from stimulated microglia [45–47]. Blockade of sodium channels with phenytoin reduced the LPS-stimulated secretion of IL-1 and TNF-α without affecting anti-inflammatory IL-10 levels in an experimental model of autoimmune encephalomyelitis (EAE), attenuating the severity of the disease [47]. In our study Rn decreased pro-inflammatory cytokine release in all range of concentrations assayed. Rn was tested at concentrations corresponding to those with therapeutic effect [13].

We also demonstrated for the first time an increase in PPAR-γ expression in primary cultured astrocytes treated with Rn. PPAR-γ is a ligand-activated transcription factor that affects the expression or activity of several genes, including those involved in the regulation of glucose homeostasis, energy metabolism and inflammation [48,49]. Evidence supports the role of PPAR-γ as anti-inflammatory factor [49]. It has been demonstrated that PPAR-γ agonists act as neuroprotective agents against neurodegenerative diseases, such as stroke [48], Alzheimer's [48,50,51] and Parkinson´s disease [48]. Additionally, Hu et al, in a mouse skeletal model, found that PPAR-γ improves cellular storage of energy and increases insulin signaling, with beneficial effects on metabolic health and tissue repair [52]. Inflammation contributes to secondary brain damage and some studies have shown that PPAR-γ reduces inflammation after ischemic and hemorrhagic stroke [53–55]. Furthermore, activation of PPAR-γ suppress NF-κB [56,57], which controls the expression of various genes involved in inflammatory responses [58]. Recent data suggest that NF-κB inhibitory pathway may stimulate N2 neutrophils phenotype with neuroprotective effects [59]. PPAR-γ also participates in the polarization of macrophages toward the M2 phenotype, which is associated with anti-inflammatory actions and tissue repair [60]. PPAR-γ agonists possess antitumor effects in combination with chemotherapy drugs or other targeted therapies and may represent a promising strategy in the treatment of malignancies [61,62]. In addition to its anti-inflammatory effect, PPAR-γ is known to upregulate Cu/Zn-SOD expression [63]. This is in line with our results where Rn significantly increased PPAR-γ and Cu/Zn-SOD expression. Zhao et al, in a model of neuron-specific PPAR-γ knockout mice, found that deficiency in neuron PPAR-γ expression leads to an increase in brain damage in response to ischemia and oxidative insult [31]. Therefore, PPAR-γ agonists might be useful to eliminate oxidative stress induced by ischemic damage and injured brain [31,64]. Antioxidant molecules, such as Cu/Zn-SOD and Mn-SOD possess the PPAR-γ positions in their promoter regions and their expressions are directly regulated by PPAR-γ [65,66]. Rn enhances glucose oxidation in different conditions, including ischemia, which in turn decreases fatty acid oxidation, improving oxygen consumption and reducing lactate production [67], thus attenuating ischemic mitochondrial damage [68,69]. In astrocytes and microglia, ROS and reactive nitrogen species are expressed in neurodegenerative diseases [70–72]. The main antioxidant defence mechanism under this pathophysiological conditions involves the activity of antioxidant enzymes such as superoxide dismutase (SOD). SOD regulates the concentration of superoxide radicals by catalysing the change of superoxide to hydrogen peroxide (H_2_O_2_) [73]. It is possible that Rn plays an important role in this mechanism since it has been reported that attenuates H_2_O_2_ release and hypoxia induced by *I*_NaL_ [74,75].

Trypan blue exclusion assay results indicate that an increase in proliferation occurs after Rn addition in a concentration-dependent manner. The mechanisms underlying proliferation and survival of astrocytes might include the MAPK signaling pathway, cell cycle regulatory molecules and microRNAs, acting independently or together to regulate astrogenesis [76]. Furthermore, factors such as ciliary neurotrophic factor (CNTF) is implicated in adult neurogenesis [77]. Astrocytes produce CNTF and express CNTF receptor α, therefore this factor could be involved in astrogenesis. It remains to be determined whether ranolazine increases astrocytes proliferation by augmenting CNTF.

Our MTT data indicate that Rn significantly increased astrocytes viability compared to control cells, without effects on cultured neurons. MTT assay is a well-established, widely used and easily reproducible method for the assessment of cell viability and cytotoxicity when cells are exposed to different substances [78,79]. Moreover, Rn decreased 15% (10^-6^M) and 20% (10^-5^M) LDH leakage in control astrocytes, suggesting less cell death [80,81]. In addition, Rn protected astrocytes from the harmful effects of Aβ_1–42_. Toxic peptide induced an significantly increased in about 75% in LDH release and incubation with Rn lowered by 60% LDH levels, indicating a protective effect against the toxic peptide. The beneficial effects of Rn on central nervous system need further studies. The effects of Rn on cell viability are probably depending on cellular type. In our experiments, cells react differently when exposed to Rn, stimulating astrocytes viability without effects on neurons. On the other hand, Rn decreased Caspase-3 activity to 25% (Rn 10^-6^M) and to 40% (Rn 10^-5^M), suggesting that Rn inhibits apoptosis in astrocytes in primary culture. Furthermore, Aβ_1–42_ increased caspase-3 activity (105%) that was reversed by Rn in 85%, showing that Rn prevents apoptosis induced by Aβ_1–42_.

Sequential activation of caspases plays a main role in cell apoptosis. Caspase 3 has critical and multiple effects leading to apoptosis, especially under specific death inducers [82]. Apoptosis process is associated with the release of Cytochrome c and Smac/Diablo proteins [83,84] from mitochondria into the cytosol in response to cytotoxic drugs or DNA damage. In this sense, Rn diminishes Cytochrome c release in guinea pig isolated hearts [6]. Smac/Diablo produces apoptosis neutralizing one or more members of the IAP family (apoptosis inhibitory proteins) [85]. Our results demonstrate a decrease in Smac/Diablo protein expression after Rn addition in astrocytes compared to control cells. Therefore, it is possible that the decrease in cell death that we found with Rn would be associated with the inactivation of the mitochondrial apoptosis pathway.

**Fig 9:**
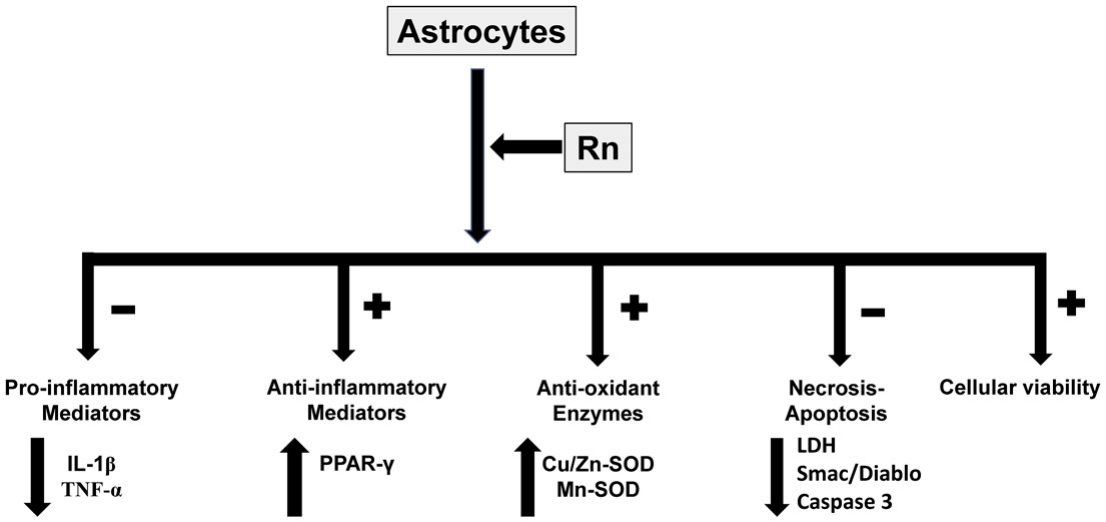
Rn effects in astrocytes in primary culture. Rn increases cell viability, anti-inflammatory response and anti-oxidant proteins. On the other hand, Rn decreases pro-inflammatory mediators and necrosis-apoptosis.

In conclusion, our results demonstrate for the first time that ranolazine induces an increase in astrocytes viability and proliferation, decreases cell death by reducing LDH release, and apoptosis by reduction of Smac/Diablo protein expression and Caspase 3 activity, diminishes pro-inflammatory mediators IL-1β and TNF-α, and increases anti-inflammatory PPAR-γ, anti-oxidant Cu/Zn-SOD and Mn-SOD protein expression in astrocytes in primary culture (Fig 9). However, none of these effects were found in neurons in primary culture. Further studies will be needed to address the role of ranolazine as a neuro-protective agent against a variety of neurological disorders, such as neurodegenerative, vascular, inflammatory, or traumatic diseases.

## Abbreviations

Rn: Ranolazine
PPAR-γ: Peroxisome proliferator activated receptor gamma
Cu/Zn-SOD: Cu/Zn superoxide dismutase
Mn-SOD: Mn superoxide dismutase
Smac/Diablo: Second mitochondria-derived activator of caspases
TNF-α: Tumor necrosis factor-α
IL-1β: Interleukin-1β
MTT: 3-(4,5-dimethyl-2-thiazolyl)-2,5-dipheniyl-2H-tetrazolium bromide
